# COVID-19 in adult patients with pre-existing chronic cardiac, respiratory and metabolic disease: a critical literature review with clinical recommendations

**DOI:** 10.1186/s40794-020-00118-y

**Published:** 2020-08-28

**Authors:** Gerard Thomas Flaherty, Paul Hession, Chee Hwui Liew, Bryan Chang Wei Lim, Tan Kok Leong, Victor Lim, Lokman Hakim Sulaiman

**Affiliations:** 1 School of Medicine, National University of Ireland Galway, Galway, Ireland; 2 National Institute for Prevention and Cardiovascular Health, Galway, Ireland; 3 School of Postgraduate Studies, International Medical University, Kuala Lumpur, Malaysia; 4 Department of Anaesthesia and Intensive Care, Cork University Hospital Group, Cork, Ireland; 5 School of Medicine, Trinity College Dublin, Dublin, Ireland; 6 Department of Emergency Medicine, Mercy University Hospital, Cork, Ireland; 7 Department of Community Medicine, School of Medicine, International Medical University, Kuala Lumpur, Malaysia; 8 Department of Pathology, International Medical University, Kuala Lumpur, Malaysia; 9 Institute for Research, Development and Innovation, International Medical University, Kuala Lumpur, Malaysia

**Keywords:** COVID-19, SARS-CoV-2, Novel coronavirus, Pandemic, Co-morbidities, Chronic illness, Travel

## Abstract

**Background:**

A high burden of severe disease and death from the coronavirus disease 2019 (COVID-19) has been consistently observed in older patients, especially those with pre-existing medical co-morbidities. The global pandemic lockdown has isolated many patients with chronic illnesses from their routine medical care. This narrative review article analyses the multitude of issues faced by individuals with underlying medical conditions during the COVID-19 pandemic.

**Methods:**

Sources for this publication were identified through searches of PubMed for articles published between 31st December 2019 and 4th June 2020, using combinations of search terms. Guidelines and updates from reputable agencies were also consulted. Only articles published in the English language were included.

**Results:**

The volume of literature on COVID-19 continues to expand, with 17,845 articles indexed on PubMed by 4th June 2020, 130 of which were deemed particularly relevant to the subject matter of this review. Older patients are more likely to progress to severe COVID-19 disease requiring intensive care unit (ICU) admission. Patients with pre-existing cardiovascular disease, especially hypertension and coronary heart disease, are at greatly increased risk of developing severe and fatal COVID-19 disease. A controversial aspect of the management of COVID-19 disease has been the use of angiotensin-converting enzyme inhibitors and angiotensin receptor blockers. Obese COVID-19 patients are more likely to require complex ICU management. Putative mechanisms of increased COVID-19 disease severity in diabetes include hyperglycaemia, altered immune function, sub-optimal glycaemic control during hospitalisation, a pro-thrombotic and pro-inflammatory state. Patients with mental health disorders are particularly vulnerable to social isolation, and this has been compounded by the suspension of non-emergency care in hospitals around the world, making it difficult for patients with chronic mental illness to attend outpatient appointments.

**Conclusions:**

The global pandemic of COVID-19 disease has had a disproportionately negative impact on patients living with chronic medical illness. Future research should be directed at efforts to protect vulnerable patients from possible further waves of COVID-19 and minimising the negative impact of pandemic mitigation strategies on these individuals.

## Background

From its presumptive origins in late 2019 at an indoor seafood and live wild animal market in the major transportation hub of Wuhan, China [1], the novel coronavirus (severe acute respiratory syndrome coronavirus 2 [SARS-CoV-2]) has spread rapidly around the world, reaching pandemic status on 11th March 2020 [2]. Travellers who departed Wuhan before the Spring Festival marking the Chinese Lunar New Year became the main source of infection for other cities in China [3]. Ultimately, international air travel accelerated the global penetration of the virus, despite the implementation of a stringent lockdown of Wuhan by the Chinese government on 24th January 2020 [4]. Seventeen years after the SARS epidemic, the coronavirus disease 2019 (COVID-19) pandemic serves as a tragic reminder of how rapidly novel pathogens can spread in a globalised world, leaving a trail of illness, death and economic hardship in their wake.

There is growing interest among the travel medicine community in assessment and mitigation of the non-communicable disease risks faced by international travellers [5]. There was a strong association in the 2012 Middle East respiratory syndrome coronavirus (MERS-CoV) outbreak between diabetes, hypertension, heart disease and death in infected patients [6]. Early reports from China during the current pandemic have also identified a particularly high burden of severe disease and death in older patients. Zhao and co-workers determined that 18.75% of COVID-19 patients had a history of chronic medical illness [7]. A meta-analysis by Wang et al. concluded that hypertension, diabetes, chronic obstructive pulmonary disease, cardiovascular disease, and cerebrovascular disease were independent risk factors for severe COVID-19 disease [8]. Onder and colleagues in northern Italy also found severe COVID-19 to be over-represented in patients with active cancer and atrial fibrillation [9]. Huang et al. showed that, among the 41 patients with SARS-CoV-2 infection in their study, 32% had underlying diseases, including diabetes in 8 patients, hypertension in 6 patients, and cardiovascular disease in 6 patients [10]. A prospective observational cohort study in the United Kingdom (UK) of 16,749 patients hospitalised with COVID-19 reported that the commonest medical comorbidities were chronic heart disease (29%), diabetes (19%), non-asthmatic chronic pulmonary disease (19%), and asthma (14%) [11]. In a meta-analysis of eight studies involving 46,248 patients infected with SARS-CoV-2, the most prevalent co-morbidities were hypertension (17 ± 7, 95% CI 14–22%) and diabetes (8 ± 6, 95% CI 6–11%), followed by cardiovascular disease (5 ± 4, 95% CI 4–7%) and respiratory disease (2 ± 0, 95% CI 1–3%). Compared with patients with non-severe disease, the pooled odds ratio of hypertension, respiratory disease, and cardiovascular disease in severe cases was 2.36 (95% CI: 1.46–3.83), 2.46 (95% CI: 1.76–3.44), and 3.42 (95% CI: 1.88–6.22), respectively [12]. In a large case series from New York, hypertension was the most common co-morbidity observed in 60% of patients with COVID-19, followed by diabetes, which occurred in 37% of the patient cohort [13]. A multivariate analysis of these data found that age, body mass index, and the presence of human immunodeficiency virus (HIV) infection or chronic kidney disease had a statistically significant association with death. A strong link has been established between black and Asian ethnicity and severe COVID-19 disease in both the UK and United States of America (USA), much of which may be at least partly explained by the predominance of underlying medical risk factors in these populations [14].

The widespread global lockdown and reconfiguration of health services to cope with the first wave of COVID-19 cases have isolated many patients with chronic illnesses from their routine medical care, leading to concerns about an anticipated surge in non-COVID medical cases as lockdown measures are gradually lifted. The rapid adoption of telehealth care has been a hallmark of the health system’s response to this pandemic [15], and the increased digital health literacy of patients is to be welcomed. The recommended domestic cocooning of older and medically vulnerable patients also raises concerns regarding lack of physical activity and its attendant cardiometabolic risks. New onset or exacerbations of pre-existing mental health conditions have also emerged as unintended consequences of the pandemic and its public health mitigation strategy. Patients with persistent medical problems may already be marginalised, with limited capacity to compensate should their condition be worsened by concomitant infection. Other determinants of ill health in this population come into play, including financial strain, housing insecurity, low pay or unemployment, and lack of access to financial and social supports.

Given the rapid growth in medical literature relating to this global pandemic, this narrative review article will focus on the issues facing adults with predisposing medical conditions, especially cardiorespiratory and metabolic, in an effort to provide greater clarity to the travelling public and guidance to their healthcare providers.

## Methods

### Literature search strategy

Two of the authors (GTF and CHL) identified the references for this review through weekly searches of PubMed between 1st May 2020 and 4th June 2020 for articles published between 31st December 2019 and 4th June 2020, using combinations of the terms “coronavirus”, “novel coronavirus”, “nCoV”, “COVID-19”, “SARS-CoV-2”, “diabetes”, “obesity”, “cardiovascular disease”, “respiratory disease”, “gastrointestinal disease”, “cancer”, “kidney disease”, “immunosuppression”, and “co-morbidities”. All articles retrieved using the general search term “COVID-19” or variations thereof were further screened by title and/or abstract for additional sources not yielded by the focused search. Articles which did not relate to at least one chronic medical comorbidity were excluded, unless they contained important general information which provided context to our narrative discussion. We reviewed guidelines for the management of COVID-19 published by the World Health Organization, the European Centre for Disease Prevention and Control, and the US Centers for Disease Control and Prevention. We also examined relevant references cited in retrieved articles. Only articles published in the English language were retrieved but the English language abstract of one Chinese language article by Peng et al. was also consulted. The final reference list was agreed by all authors on the basis of its relevance to the topics covered in this review, with the aim of exploring the clinical presentation and complications of COVID-19 in patients with co-morbid conditions, and in presenting authoritative guidance to practitioners who manage patients with these chronic illnesses.

## Results

The volume of literature on COVID-19 continues to expand at a rapid rate, with 17,845 associated articles indexed on the PubMed database alone as of 4th June 2020. After screening titles, abstracts and, in some cases, the full text version of indexed articles, 130 articles were deemed relevant to the subject matter of this review.

### Older patients with co-morbid conditions

The rapid global spread of the SARS-CoV-2 infection has challenged traditional efforts to communicate medical information in a timely manner to older patients living with chronic illness. The majority of older travellers attending a specialist travel clinic in Ireland were found to have a pre-existing medical condition [16]. In the early stages of this pandemic, many adults with co-morbid diseases were poorly aware of COVID-19 and had not adapted their routines or self-management plans [17]. Community-based patient organisations and support groups have since played a leading role in mobilising and educating the most vulnerable patients. There has been particular attention given in the recent medical literature to the impact of the pandemic on the older population.

Older patients have borne a disproportionate burden of severe disease and death from COVID-19. Elderly patients with COVID-19 are more likely to progress to severe disease requiring intensive care unit (ICU) admission [18]. In a case series of 138 hospitalised patients with COVID-19 pneumonia, the median age of patients transferred to the ICU was 66 years, compared to 51 years in patients not receiving ICU care. Co-morbidities were present in 72.2% of cases, while only 37.3% of non-ICU COVID-19 patients had at least one co-morbid condition [19]. The case-fatality rate for COVID-19 in one study from the USA was found to be 10–27% in patients over the age of 85 years [20]. Patients aged 65 years and above accounted for 45% of hospitalisations, 53% of admissions to the ICU, and 80% of all deaths from the infection. Patients over the age of 70 years had shorter median time intervals between symptom onset and death (11.5 days) when compared with younger adults (20 days) [21]. In a multicentre, retrospective study of 105 elderly patients with confirmed COVID-19 in Hunan Province, China, 69.5% of the elderly patients had underlying conditions, with hypertension (43.8%), diabetes (25.7%), and cardiac disease (16.2%) as the most common co-morbidities. Of the elderly patients, 22.9% had severe COVID-19 disease and 10.5% were critically ill [22].

The devastating effect of COVID-19 clusters in residential care settings has been a tragic feature of this pandemic. Older people living in residential care facilities have been over-represented among the victims of this infection, and there has been much discussion about the adequacy of the public health response in safeguarding older residents by ensuring that carers have access to personal protective equipment. People living with dementia have been particularly marginalised, given the difficulties they face in registering and recalling safety advice, such as the wearing of barrier face masks, or in processing the public health messages relayed by the media [23]. This places them at particular risk when self-quarantine measures are imposed. Community volunteer efforts have helped to offset the negative effects on the elderly of prolonged lockdown advisories, by helping with the delivery of food and essential supplies during quarantine [24]. The frequent occurrence of delirium in hypoxic COVID-19 patients further complicates efforts to orientate patients with dementia [25]. One of the most poignant aspects of the pandemic has been the dichotomisation of COVID-19 patients according to age when critical care teams, at a time of increased stress on health systems, are inevitably faced with triage decisions about which severely ill patients should be mechanically ventilated in ICUs [26]. Indeed, this pandemic has imposed limitations on access to ICU care for all patients with complex or persistent non-COVID illnesses based on the scarcity of ICU resources in most countries.

### Patients with chronic respiratory disease

Despite earlier suggestions that cigarette smoking, by up-regulating the expression of angiotensin-converting enzyme 2 (ACE-2) receptors in distal airways, may be responsible for causing more severe COVID-19 disease [27], a subsequent meta-analysis failed to observe a statistically significant association between active smoking and COVID-19 severity in four out of five studies reported [28]. The ultimate impact arising from the cancellation of routine in-person addiction services, such as smoking cessation clinics, merits formal evaluation. Asthma is regarded as a risk factor for COVID-19 morbidity and this is likely to be secondary to the tendency of SARS-CoV-2, like other respiratory viruses, to trigger asthma exacerbations [29]. Patients with asthma have been advised to remain on their regular maintenance therapy during the pandemic and to review their inhaler technique [29]. Nebulisers should be used with caution, particularly in health care settings. Dry powder inhalers or metered-dose inhalers with a valved holding chamber are preferred delivery methods [30]. Oral steroids, though generally not recommended in the treatment of COVID-19 lung injury [31], are still acceptable for use in patients with moderate to severe exacerbations of asthma that fail to respond to bronchodilators [32].

Patients with cystic fibrosis (CF) who develop COVID-19 lung disease must contend with the risk of suffering a negative effect on lung function [33], and they should have a low threshold for undergoing COVID-19 viral polymerase chain reaction testing. Cancellation of routine outpatient appointments has led to a greater use of telemedicine with self-monitoring of spirometry and pulse oximetry data where patients have been suitably trained; these tele-clinics are less likely to have an interdisciplinary focus, however. It is likely that CF patients will suffer from a reduced access to registered clinical trials and to lung transplantation services during the pandemic [34]. Both obstructive sleep apnoea and obesity hypoventilation may worsen the hypoxaemia in patients with COVID-19 lung disease [35, 36]. The effects of obesity on the course of COVID-19 will be considered in greater detail later in this review.

### Patients with cardiovascular disease

Patients with pre-existing cardiovascular disease (CVD), especially hypertension and coronary heart disease, are at greatly increased risk of developing severe and fatal COVID-19 disease. A meta-analysis of six studies with 1527 patients identified that hypertension and cardio-cerebrovascular disease were present as co-morbidities in 17.1 and 16.4% of the patients, respectively [37]. In their study of risk factors for adult inpatient deaths from COVID-19, Zhou et al. recorded a 45% death rate in patients with arterial hypertension, which greatly exceeded the 28% death rate in the entire case series [38]. In the same study, 86% of the patients with coronary heart disease died from COVID-19. The Chinese Center for Disease Control and Prevention reported a case-fatality rate of 6.0% for patients with hypertension and 10.5% for CVD patients, against a background case-fatality rate of 2.3% among their 44,672 confirmed COVID-19 cases [39]. According to a retrospective analysis of 2877 hospitalised patients, 29.5% had a history of hypertension which, after adjusting for confounders, conferred a two-fold increased relative risk of death compared with normotensive patients (adjusted hazard ratio 2.12, 95% CI 1.17–3.82) [40]. A prospective cohort study of 179 patients with COVID-19 pneumonia determined that concurrent cardiovascular or cerebrovascular disease were associated with an increased risk of death (odds ratio 2.464, 95% CI 0.755–8.044; *p* = 0.007) [41].

Potential mechanisms of myocardial injury in COVID-19 include viral entry via the ACE-2 receptor, hypoxia-induced myocyte damage, and an immune-mediated cytokine storm [42]. Recent data have demonstrated a marginal increase in cardiac troponin I concentrations in patients with SARS-CoV-2 infection [43]. It has been suggested that measurement of these myocyte injury biomarkers initially in infected patients, and at intervals during their hospital admission, may allow stratification of patients according to their risk of cardiac injury and therefore the risk of more severe COVID-19 disease. N-terminal pro-B-type natriuretic peptide measurement has also been suggested as an independent risk factor for in-hospital death in patients with severe COVID-19 [44].

While the positive environmental effects of the societal lockdown, in terms of reduced particulate air pollution and diminished traffic noise, are likely to have an appreciable population-level cardioprotective effect [45], these benefits could be out-weighed by a variety of negative socioeconomic consequences, including unemployment, personal stress, depression, anxiety and isolation from social contacts [46], all of which are considered potent risk factors for atherosclerotic cardiovascular disease.

A controversial aspect of the management of COVID-19 has been the use, in some patients with hypertension, of ACE inhibitors (ACEI) and angiotensin receptor blockers (ARB). ACE-2 receptor upregulation in infected patients taking these antihypertensive agents is believed to generate higher viral loads [47]. This is countered by the theory that increased ACE-2 receptor expression, by increasing the concentration of ACE-2 in the blood, may bind to the virus and prevent its interaction with the cell membrane bound receptor [48]. A retrospective analysis of 112 COVID-19 patients with CVD in Wuhan, China found no significant difference in the use of ACEI/ARB in those that survived or succumbed to the disease [49]. A further concern is the risk of medical errors in hypertensive patients should their ACEI/ARB be discontinued, since these patients would have to attend their family doctor and pharmacy and undergo frequent dose titration and management of adverse effects from alternative drugs [50]. This therapeutic complexity should be minimised where possible during a pandemic situation. Mortality rates were similar between hypertensive patients with COVID-19 taking renin-angiotensin-aldosterone system (RAAS) inhibitors and the non-RAAS inhibitor cohort in a large recent study from China [40]. The Council on Hypertension of the European Society of Cardiology, having appraised the risk-to-benefit ratio of RAAS blockers in hypertensive patients, have recommended against their discontinuation in patients with COVID-19 [51].

### Obesity and COVID-19

The challenges faced by obese international travellers have attracted recent attention in the medical literature [52]. Obese COVID-19 patients report more severe symptoms than normal weight patients and they are more likely to require complex ICU management, including invasive mechanical ventilation [53]. In a study of 3615 patients from New York City, being obese (BMI 30–34 kg/m^2^) and under the age of 60 years predicted an approximately two-fold increased risk of requiring acute and critical care [54]. This risk was further increased at higher levels of body mass index. Suggested mechanisms for the greater clinical severity of COVID-19 in obese patients include a reduced end-expiratory volume, positive pleural pressures at end-exhalation [36], chronic low-grade inflammation [55], and an altered immune response to infection [56]. There is a much higher incidence of type 2 diabetes in this cohort of patients, and this acts as an additional risk factor for severe COVID-19 disease.

The effects of prolonged societal lockdown measures on obese individuals’ attempts at weight management also deserve attention. It has been suggested that obese subjects may require a longer period of quarantine than normal weight individuals, owing to their prolonged viral shedding [57]. Strategies to prevent progression of obesity during lockdown, including caloric restriction and mild-moderate physical activity, have been advocated by a leading professional obesity organisation [58]. Obese patients face considerable weight bias, both in health care settings and during international travel [59]. The circulation of fat-shaming memes on social media during the current pandemic lockdown should therefore be condemned by health care professionals. Supportive obesity care, which includes motivational interviewing techniques, should be delivered by telemedicine approaches until routine outpatient health services have resumed. The negative impact of the pandemic on access to elective bariatric surgery waiting lists remains to be determined.

### Patients living with diabetes

Diabetes mellitus has rapidly become established as a major co-morbidity for severe COVID-19 disease [60]. Patients with diabetes have up to a 50% greater chance of a fatal outcome from COVID-19 than non-diabetic infected individuals [61]. Increased mortality in this cohort was also observed in the two previous coronavirus pandemics caused by infection with SARS and MERS-CoV [62]. It is believed that diabetic individuals are not more susceptible to developing viral infection per se but are more likely to show increased clinical severity and to require ICU admission. In hospitalised COVID-19 patients with diabetes, body mass index, but not long-term glycaemic control, was independently associated with tracheal intubation and/or death within 7 days of hospitalisation [60]. Putative mechanisms of disease severity in diabetes include hyperglycaemia, altered immune function, sub-optimal glycaemic control during hospitalisation, reduced forced vital capacity and forced expiratory volume in 1 s on lung function testing, as well as a pro-thrombotic and pro-inflammatory state [63, 64]. The frequency of fatty liver disease in type 2 diabetes patients may put them at increased risk for an exaggerated inflammatory response including development of a cytokine storm, which is associated with severe COVID-19 lung injury [64].

This group of patients benefits from the renoprotective effect of RAAS blocking antihypertensive agents. As discussed earlier, it is currently recommended that ACEI and ARB be continued in COVID-19 patients. Continuous blood glucose monitoring is recommended in hospitalised COVID-19 patients with diabetes, with use of sliding scale insulin therapy [65]. The possible role of dipeptidyl peptidase 4 (DPP-4) as a receptor for SARS-CoV-2 raises the potential for DPP-4 inhibitors in diabetic COVID-19 patients, but further research is needed to investigate their role [66]. It is recommended that metformin and sodium-glucose cotransporter 2 inhibitors should be discontinued in diabetic patients with severe COVID-19 disease to reduce their risk of developing lactic acidosis or diabetic ketoacidosis, respectively [64].

In common with other chronic illness populations, diabetic patients should have access to telemedicine services in order to benefit from the continuity of routine primary or outpatient care during the pandemic lockdown period. This may be supplemented by the transmission in real-time of capillary blood glucose readings to the diabetes health care team using remote sensor technology. Lifestyle advice which encompasses physical activity should be provided by members of the multidisciplinary diabetes health care team. A summary of practical clinical guidance for diabetes management during the current pandemic is presented in Table 1.

**Table 1:** Clinical recommendations for management of diabetes during COVID-19 pandemic (modified from Bornstein et al. [64])

Clinical Setting	Therapeutic Goal	Recommendations
Outpatient/community	Prevent infection	Educate patients about importance of glycaemic control Optimise current management Do not discontinue existing therapy Use Connected Health to maintain contact with patient
	Optimise glycaemic control	Aim for plasma glucose of 4–8 mmol/L and HbA_1c_ < 53 mmol/mol (7%) Hypoglycaemic (< 3.9 mmol/L) less than 4% (< 1% in frail/elderly) Encourage moderately intense physical activity
In-patient/ ICU	Monitor for new onset of diabetes	Plasma glucose monitoring Measure venous blood pH Check blood ketones
	Manage infected patients	Low threshold for early IV insulin in severe cases (e.g. ARDS)
	Achieve glycaemic control	Aim for plasma glucose of 4–10 mmol/L

*ICU* Intensive care unit, *IV* Intravenous, *ARDS* Acute respiratory distress syndrome

### Chronic kidney disease and haemodialysis

Meta-analysis has demonstrated a significant independent association of chronic kidney disease (CKD) with severe COVID-19 [67]. The use of immunosuppressant drugs in patients who have received a renal transplant is of obvious concern during a pandemic as these agents expose transplant recipients to a higher risk of infection. It has been recommended to only aggressively reduce the dose of maintenance immunosuppressive therapy in CKD patients with severe COVID-19 or in those who have developed acute respiratory distress syndrome (ARDS) [68]. Curtailed organ-sharing across dispersed geographical locations and a diminished organ donor pool will limit access to solid organ transplantation and heighten the burden of end-stage renal disease, leading ultimately to a greater number of patients dying on transplant waiting lists. The issues surrounding the containment of infection in dialysis units has been discussed in the recent nephrology literature and home dialysis has been promoted as a viable alternative where this service can be supported [69]. Table 2 summarises the clinical recommendations for renal dialysis patients and their health care providers.

**Table 2:** Reduction of COVID-19 transmission in haemodialysis centres (based on Basile et al. [69])

Target Group	Recommendation
Healthcare professionals	Receive training in use of personal protective equipment Inform healthcare team leader if symptomatic or contact with known case Self-isolate if unwell Use full PPE when caring for confirmed cases of COVID-19
Renal dialysis patients	Observe hand hygiene on arrival and departure from unit Observe respiratory etiquette Wear a suitable barrier face covering Have body temperature checked before and after session Inform staff if symptomatic before arrival at unit Be educated on how to self-isolate If symptomatic/diagnosed case should undergo dialysis in a separate isolation room

### Cancer and COVID-19

The volume of calls to a leading UK-based cancer charity during the COVID-19 pandemic lockdown reflects the understandable anxiety among patients undergoing treatment for, or recovering from, cancer [70]. It is not yet clear if cancer patients have an increased risk of developing COVID-19 [71], but they are likely to be highly susceptible to adverse outcomes by virtue of being severely immunocompromised from both their cancer and its therapy [72]. In a study by Liang and colleagues, 18 of 1590 patients with COVID-19 had a history of cancer [73]. Patients with cancer were found to have a higher risk of requiring ICU-based mechanical ventilation or dying from COVID-19 [73]. The risk was especially pronounced for patients who had undergone chemotherapy or surgery in the month prior to admissions. Chemotherapy is a recognised risk factor for infection, although there has been a suggestion that the blunted immune response it causes may generate a less pronounced cytokine storm [71], which may be of benefit to cancer patients.

Deferral of adjuvant chemotherapy or elective surgery for stable cancer patients has been advocated during the pandemic [73]. It has been recommended that surveillance using laboratory investigations and imaging should be postponed for several months in asymptomatic cancer patients [73]. A prolonged period of cocooning has been advised for vulnerable cancer patients undergoing active cancer treatment [70]. Concerns have been expressed by oncologists about the delays in diagnosing and treating cancer during the current lockdown phase. The survival benefit of hospital admission for existing cancer patients must be carefully weighed against the risk of contracting COVID-19 during hospitalisation [74]. Increasingly, protocols for formal SARS-CoV-2 screening of patients awaiting elective cancer chemotherapy, radiotherapy or surgery are being developed, but anecdotally the lack of clear guidelines or consensus on how to proceed when virus is detected in asymptomatic patients is generating delays in treatment and contributing to increased morbidity in this vulnerable patient group.

Jindal et al. concluded in their recent guidance that, notwithstanding the potential risk of infection at chemotherapy day wards, chemotherapy should be continued with stringent measures taken to prevent transmission of the virus [72]. This includes keeping patients on chemotherapy in an isolation unit or in self-isolation at home for at least 7 days before commencing chemotherapy. How immune checkpoint inhibitors used in advanced cancers will influence the outcome of SARS-CoV-2 infection and its treatment is currently unknown. It is recommended that non-urgent bone marrow transplantation (BMT) be deferred and that patients and the families of patients who recently received a BMT should follow strict hygiene precautions to prevent exposure to SARS-CoV-2 [72].

### Rheumatic disease and immunosuppressed patients

Patients with a compromised immune system are anticipated to be at risk of more severe COVID-19 disease. A study by Sawalha et al. suggested that epigenetic dysregulation of the ACE-2 gene may confer an increased susceptibility and severity of COVID-19 disease in patients with systemic lupus erythematosus [75]. There is no contraindication to the use of anti-inflammatory drugs or disease modifying anti-rheumatic drugs during the COVID-19 pandemic [76]. Despite early enthusiasm for its use, hydroxychloroquine is no longer considered to be an effective therapy for patients with COVID-19 [77]. Patients who take maintenance immunosuppressive therapy for management of psoriasis or rheumatic diseases such as rheumatoid arthritis (RA) should follow the advice not to discontinue their use of biologic agents prematurely [78]. Tumour necrosis factor-alpha inhibitors are not believed to increase the risk of viral infection but may have a therapeutic effect in patients with COVID-19 [79]. Intra-articular corticosteroid injections are considered acceptable in the absence of a reasonable therapeutic alternative, with the provision that all precautions are taken to protect the patient and medical professional from viral contamination [76]. Recommendations for commencing or discontinuing immunosuppressive therapy in patients with psoriasis are summarised in Table 3.

**Table 3:** Recommendations for use of biologic therapy in psoriasis (after Nogueira et al. [78])

Management Issue	Recommendation
Risk of severe COVID-19	Modest increased risk of URTI with TNF-alpha, IL-12/23, IL-23 and IL-17 blockers Immunomodulatory drugs used for treatment of COVID-19 infection Evaluate risk-to-benefit ratio for each patient Do not blanket suspend biologic agents in all patients with psoriasis
Risk of flare-up of psoriasis	Flare-ups may necessitate visit to clinic or hospital with risk of COVID-19 transmission Suspend biologic agent only for proven COVID-19 patients until fully recovered Consider screening for SARS-COV-2 in patients commencing biologic therapy
Risk of drug resistance	Suspension and reintroduction of biologics may generate antibodies that affect response to drug, especially for TNF-alpha inhibitors

*URTI* Upper respiratory tract infection, *TNF* Tumour necrosis factor, *IL* Interleukin

Qualitative analysis of participants in a US-wide longitudinal observational registry revealed that 42% of patients with RA experienced a disruption to their normal treatment in the preceding 2 weeks [80]. The following themes emerged from the study: the emotional response to the pandemic; perceptions of risk from immunosuppressive medications; measures to reduce personal risk of contracting COVID-19; and difficulty accessing anti-rheumatic drugs, including hydroxychloroquine.

Patients living with HIV infection suffer from more respiratory illnesses than HIV-negative individuals [81]. Case series of COVID-19 in patients living with HIV are lacking. One percent of admissions to a Spanish hospital had HIV and clinical outcomes were generally favourable in this group [82]. The authors suggested that boosted-protease inhibitor anti-retroviral therapy may have activity against the coronavirus protease, thus conferring a degree of protection from severe disease in patients living with HIV.

### Gastrointestinal disease and COVID-19

The finding of prolonged shedding of SARS-CoV-2 viral ribonucleic acid in stool [83] has raised the possibility of faecal-oral transmission of COVID-19, via contaminated food or as fomite spread in the toilet flush plume [84]. Abdominal symptoms, including nausea, diarrhoea and abdominal pain, have been reported in a minority of patients with the disease [85]. Patients with COVID-19 frequently display raised liver transaminases and hypoalbuminaemia, the latter being suggested as a diagnostic marker [86]. Mechanisms of liver injury in COVID-19 have been discussed comprehensively elsewhere [87]. The British Society of Gastroenterology has issued guidelines regarding the use of medications in patients with inflammatory bowel disease during the COVID-19 pandemic (Table 4) [89]. Patients are advised not to discontinue their current active treatment. They should also have access to telemedicine services where attendance at outpatient clinics is not possible.

**Table 4:** Guidelines for patients with inflammatory bowel disease during COVID-19 pandemic (adapted from Mao et al. [88])

Management Priority	Recommendation
Risk factors for SARS-CoV-2 infection	Patients taking immunosuppressive agents Malnourished patients with active IBD Elderly IBD patients IBD patients with comorbid medical conditions Pregnant IBD patients
Drug therapy of IBD	Continue current therapy if disease is stable Continue use of mesalamine, corticosteroids, anti-TNF-alpha biologic agents (e.g. infliximab) Consider use of enteral nutrition if biologics not accessible Avoid commencement of tofacitinib unless no alternatives are available
Endoscopy and surgical procedures	Defer endoscopy and elective surgery Screen for COVID-19 by nucleic acid detection and chest CT before emergency surgery
Symptomatic IBD patients	Contact specialist team about option to attend outpatient clinic with use of PPE if temperature remains > 38 °C If suspected diagnosis of COVID-19, suspend use of immunosuppressants and biologic agents after consulting own gastroenterologist

*PPE* Personal protective equipment

### Patients with mental health disorders

A cross-sectional study found a much higher prevalence of depression (48.3, 95% CI: 46.9–49.7%), anxiety (22.6, 95% CI: 21.4–23.8%), and anxiety-depression (19.4, 95% CI: 18.3–20.6%) during the COVID-19 outbreak in Wuhan, China [90]. The COVID-19 pandemic and its mitigation measures have exacted a heavy toll on the global population’s mental health, with prolonged periods of lockdown leading to isolation from family and friends. This has been particularly marked for older individuals, who have been advised to cocoon in their homes for extended periods of time. Remote working practices have imposed a major strain on many employees, who find themselves cut off from the support and camaraderie of their colleagues. The social burden on children who are being home-schooled has led to public calls for an accelerated lifting of lockdown restrictions. There is a growing recognition of the risk of post-traumatic stress disorder in front-line healthcare workers, the manifestations of which may not be apparent for some time to come.

Pandemics tend to engender anxiety and depression, and lockdown is recognised as a cause of insomnia [91]. The psychological morbidity associated with the last SARS pandemic has been well documented [92]. The current pandemic has been marked, especially in its early stages, by a so-called ‘infodemic’ of misinformation from online platforms, including social media, which have served to amplify people’s fears [93]. The World Health Organization helped to counter this parallel pandemic of fear by publishing information on its website, which it hopes will help to dispel some of the myths surrounding the illness [94].

Patients with mental health disorders are particularly vulnerable to social isolation. This has been compounded by the effective suspension of non-emergency care in hospitals around the world, making it difficult for patients with chronic mental illness to attend outpatient appointments, for example. Some patients with psychosis face healthcare inequalities relating to a lack of a sense of self-protection and difficulty adhering to self-isolation guidelines [95].

The importance of safeguarding access to clozapine monitoring clinics and depot antipsychotic injections for patients with chronic schizophrenia has been highlighted by the psychiatric profession [96]. While there are currently no data on the COVID-19 in clozapine-treated patients, clinicians should be vigilant for signs of infection in these patients [97]. Delirium is a common, but oft overlooked, clinical manifestation of COVID-19. Clinicians should be aware that the SARS-CoV-2 coronavirus is neurotropic and that infected patients with chronic schizophrenia are at risk of psychotic relapse [98].

A particularly distressing aspect of this pandemic has been the increase in racist attitudes towards people of presumed Chinese ethnicity, given the probable origin of the novel coronavirus in Wuhan, China [93]. As the pandemic has progressed, these earlier attitudes appear to have dissipated somewhat, but negative comments continue to be directed at so-called geographic hotspots of infection. Furthermore, the Black, indigenous and people of colour (BIPOC) community face particular challenges during this pandemic, arising from an increased manifestation of severe COVID-19 disease and death, barriers to accessing medical care and over-representation among the essential workforce, thus placing BIPOC individuals at greater risk of exposure to SARS-CoV-2. The aforementioned challenges to the BIPOC community in this pandemic can be largely traced directly or indirectly to systemic and structural racism. These factors combine to significantly undermine health and resilience to severe disease in this population.

Increasing levels of alcohol misuse during lockdown are also of concern and this has contributed to an increase in levels of reported domestic abuse. The potential public health effects of long-term isolation on rates of alcohol misuse are largely unknown [99], but there have been calls for governments to issue public health warnings about the specific dangers of alcohol misuse during lockdown. The practice of online ordering of alcoholic beverages for takeaway or delivery has also attracted criticism on public health grounds.

### Male pattern baldness and COVID-19 severity

Male pattern baldness, though not generally regarded as a serious chronic medical condition, does predict a higher risk of developing coronary heart disease, hypertension and prostate cancer. An intriguing recent observation that patients being treated with hormonal therapy for prostate cancer showed a degree of protection against severe COVID-19 disease has led to suggestions that androgenetic alopecia may be a risk factor for severe infection with SARS-CoV-2. This has been invoked as a plausible explanation for why adult males develop more severe disease than females [100], why prepubescent children have been largely spared, and why there is a higher incidence of severe COVID-19 in members of the African-American community [101]. Both the ACE-2 receptor and the TMPRSS2 transmembrane protease are involved in mediating entry of SARS-CoV-2 into type II pneumocytes. Both are affected by male sex hormones, with higher activity found in males [102]. It has been hypothesised that anti-androgen therapy may be used as a treatment to reduce the risk of developing severe SARS-CoV-2 infection and this is currently being investigated [100]. The high incidence of androgenetic alopecia among patients hospitalised with severe COVID-19 has gained the eponymous description of “Gabrin’s sign”, in memory of Dr. Frank Gabrin, the first American physician to die as a result of severe SARS-CoV-2 infection [103]. While the putative mechanism underlying this association is of interest, we acknowledge that prospective research will be needed to accurately test this hypothesis.

## Discussion

To the best of our knowledge, this is the first review article addressing the impact of the COVID-19 pandemic on patients with chronic medical conditions. We have attempted to critically discuss the issues relating to a wide range of common and important medical illnesses but our coverage cannot be considered exhaustive and relevant disorders which have been omitted from this work may be the subject of future reviews on the subject. A summary of the major issues relating to COVID-19 in patients with long-term illnesses is presented in Table 5.

**Table 5:** Principal issues affecting patients with comorbidities during COVID-19 pandemic

Health issue	Comorbidities	Impact of COVID-19
Predisposition to severe disease	Obesity	More severe symptoms of COVID-19; requirement for complex ICU management; poorer clinical outcomes
	Diabetes	Increased clinical severity and mortality
Access to routine medical care	COPD	Cancellation of smoking cessation clinics
	Cystic fibrosis	Decreased access to multidisciplinary care and transplantation
	Cancer	Necessity to defer chemotherapy in some cases
	Chronic kidney disease	Infection risk in dialysis units; decreased access to renal transplantation
	Mental health disorders	Barriers to accessing outpatient care
Influence of chronic medications	Hypertension	Controversy around use of RAAS blockers
	Autoimmune disease	Immunosuppressant drugs predispose to infection
	Chronic kidney disease	Immunosuppressant drugs predispose to infection
Negative effects of pandemic lockdown	Obesity	Restrictions on physical activity; excessive caloric intake
	Mental health disorders	Social isolation; heightened anxiety; sleep disturbance; reduced capacity of psychotic patients to adhere to public health advice

*ICU* Intensive care unit, *COPD* Chronic obstructive pulmonary disease, *RAAS* Renin-angiotensin-aldosterone system

### Study limitations

The volume of new publications relating to COVID-19 continues to increase at a rapid rate. As such, this narrative literature review may not retain its currency for long. At the time of writing, there was still a paucity of original studies specifically addressing the issues facing patients with chronic medical illness, and some of the sources represented published editorial and expert consensus material. Future reviews should build on our current knowledge and provide updates during this dynamic period based on a larger proportion of research articles and meta-analyses, which provide a higher level of evidence. Our review was largely restricted to articles published in the English language, and potentially relevant studies published in Mandarin Chinese and other languages may have been omitted for consideration.

### Future directions and research priorities

This review has raised important questions, which should be addressed in future research grant calls. The effects of prolonged societal lockdown on cardiovascular and metabolic health parameters will help to guide future public health strategy and avoid an increased burden of preventable non-communicable disease. Whether certain vulnerable socio-economic groups were particularly disadvantaged, as suggested by recent reports of increased COVID-19 burden in ethnic minority groups in the UK and USA [14], is worthy of deeper consideration. The disruption to normal face-to-face chronic illness management has been mitigated to some degree by the greater use of telemedicine, but the long-term effects of this intervention deserve to be investigated. The reluctance of patients to attend hospitals during a pandemic involving a very infectious virus also merits further study. How health services will cope with the increased volume of patients on hospital outpatient waiting lists remains to be seen. The effects of deconditioning of cocooning older patients on their mobility and falls risk may also present challenges for their carers. How such unintended consequences of lockdown can be avoided in future phases of this and other pandemics should be the subject of research.

As countries take tentative steps towards the relaxation of stringent border control measures, the future landscape of international travel is being hotly debated [104]. Inevitably, travellers with pre-existing conditions will face uncertainty about their medical fitness to fly in particular until, or if, an effective vaccine against SARS-CoV-2 has been developed. Already, clearer guidance is emerging about the use of barrier face masks to protect medically vulnerable individuals in shops or on public transport, where physical distancing cannot be assured. Pre-travel health advice for patients with pre-existing cardiovascular disease [105] and diabetes will need to be updated to reflect new guidelines. A more tailored approach to performing pre-travel health risk assessments will be warranted [106]. Moreover, the attitudes and concerns of patients with chronic medical illness towards the gradual resumption of international travel should be urgently explored. Positive traveller health behaviours are likely to emerge from this pandemic, including a diminished seasonal influenza vaccine hesitancy and a greater personal focus on hand hygiene and respiratory etiquette, which may serve to reduce the future burden of respiratory and diarrhoeal illness in international travellers.

## Conclusions

The global pandemic of COVID-19 disease has had a disproportionate impact on patients living with chronic medical illness. Underlying hypertension, cardiovascular disease, diabetes, obesity and respiratory disease have emerged as significant risk factors for development of severe COVID-19 pneumonia and systemic inflammation. Patients who are immunosuppressed by virtue of their chronic disease or its therapy have also been identified as vulnerable groups. The realignment of health services towards providing emergency and critical care for large numbers of COVID-19 patients has compromised the delivery of routine care to patients with chronic diseases. Lockdown measures have also negatively impacted the metabolic health of many patients by limiting opportunities for physical activity. Travel restrictions and media saturation with COVID-19-related information have also challenged the mental health of some individuals, especially those with underlying mental illness or its risk factors. Future research should be directed at efforts to protect vulnerable patients from possible further waves of COVID-19 and to minimise the negative impact of pandemic mitigation strategies on these individuals.

## Data Availability

All material referenced in the preparation of this work are available from the corresponding author.
